# Effects of Integrative Neuromuscular Training Combined With Regular Tennis Training Program on Sprint and Change of Direction of Children

**DOI:** 10.3389/fphys.2022.831248

**Published:** 2022-02-10

**Authors:** Zhi-Hai Wang, Rui-Cheng Pan, Meng-Ru Huang, Dan Wang

**Affiliations:** School of Physical Education and Sport Training, Shanghai University of Sport, Shanghai, China

**Keywords:** sensitive period, physical fitness, movement, performance, child

## Abstract

**Objective:**

The aim of this study was to investigate the effects of integrative neuromuscular training (NMT) on sprint and the ability to change direction for children who are between the ages of 7 and 8 and beginning to play tennis.

**Methods:**

Thirty-two participants were randomized into a training group (TG; *n* = 16) and a control group (CG; *n* = 16). All participants attended tennis classes twice a week for a continuous 8 weeks. In addition, the TG received NMT (e.g., 20-m sprints, running at four corners, rope ladder drills, etc.), which progressed in difficulty every 2 weeks. Pre-intervention and post-intervention measurements, including a 30-m sprint test, a 5–10–5 test, and a 3 × 10 m shuttle run test, were assessed by a Smartspeed laser timing gate system, while the spider agility test was evaluated with a stopwatch.

**Results:**

Two-way repeated measures ANOVA found significant differences in the interaction between time and group among variables measured. Results were as follows: time in the 30 m sprint (*F* = 13.467, 95% CI = 7.163–7.506, *p* = 0.001, *η*^2^*_p_* = 0.310, Δ = 0.42 s); 5–10–5 test (*F* = 13.975, 95% CI = 8.696–9.017, *p* = 0.001, *η*^2^*_p_* = 0.318, Δ = 0.78 s); 3 × 10 m shuttle run (*F* = 7.605, 95% CI = 11.213–11.642, *p* = 0.01, *η*^2^*_p_* = 0.202, Δ = 0.77 s); and spider agility test (*F* = 34.555, 95% CI = 28.258–29.670, *p* < 0.001, *η*^2^*_p_* = 0.535, Δ = 3.96 s). The results demonstrated a greater decrease in sprint and change of direction (COD) time among the TG than the CG from pre-intervention to post-intervention.

**Conclusion:**

A regular tennis training combined with NMT program could produce greater improvement in a player’s sprint and ability to change direction when introduced to childhood tennis beginners in a sensitive period, compared to tennis class intervention only.

## Introduction

Tennis is a high-intensity and demanding sport, requiring players to repeatedly engage in a sequence of intense activities, such as accelerations, decelerations, COD, and strokes during a variable period of competition time (on average 90 min; Fernandez-Fernandez et al., 2017). Players commonly complete more than 1,000 CODs in each competitive match (Kovacs, 2009). Tennis players must move an average distance of 8–15 m, complete four COD movements, and hit the ball an average of 4–5 times for every one point scored (Murias et al., 2007). Typically, players must move quickly in linear directions (i.e., acceleration) and react quickly in both lateral and multiple directions (Fernandez-Fernandez et al., 2014). This requires attaining a high level of speed and COD ability (Fernandez-Fernandez et al., 2014). A previous study showed that speed and COD are decisive factors for tennis performance, and maximizing such abilities promotes on-court success (Fernandez-Fernandez et al., 2014). The 30 m sprint, 3 × 10 m shuttle run, spider agility, and 5–10–5 tests have been widely used to assess tennis players’ speed and COD ability (Kramer et al., 2016; Rossi et al., 2017; Cheng, 2018; Vigh-Larsen et al., 2021). The 30 m sprint and 3 × 10 m shuttle run tests assess a tennis player’s ability to accelerate in a straight line and run forward and backward from baseline to net, respectively. The combination of the two tests provides a more comprehensive assessment of a tennis player’s speed ability than either alone. The spider agility and 5–10–5 tests assess different routes of movement in a tennis player’s ability, such as lateral, forward, and backward, as well as the ability to move sideways along the back line of the court (known as the baseline). Combining these two tests provides a comprehensive assessment of a tennis player’s full-court COD ability. Given the good neuroplasticity of 7–8 years old, training related to neural adaptation may be beneficial (Rumpf et al., 2012; Lloyd et al., 2013). Generally, sprint and COD training for children (7–8 years old) is effective and safe (Lupo et al., 2019). For example, Alonso-Aubin et al. (2021) reported significant improvements in dominant and non-dominant hand-eye coordination, core muscular endurance and sprinting ability in rugby players aged 6–14 years after 8 weeks of NMT (including sprint training, etc.). Similarly, Michailidis et al. (2019) conducted a 1-year sprint training intervention (including soccer training program) for school children aged 8–10, and found that the scores of anthropometries (e.g., body weight and body fat et al.), 30 m sprint and standing long jump were significantly improved. As for the trainability of COD during childhood, directly related literature are sparse. However, the Youth Physical Development model suggests the need for COD training (Lloyd et al., 2013) throughout childhood and adolescence (Lloyd and Oliver, 2012). Lloyd et al. (2013) considered that both children and adolescent athletes should receive training program including fundamental movement skills, COD and reactive agility training, but the percentage of time should be allocated according to different stages (e.g., 25% COD training in prepubertal stage).

The term “sensitive period” was originally used by Dutch biologist Hugo de Vries in his studies of animal growth. At the beginning of the last century, the Italian physician Maria Montessori, during her long-time work with children, discovered certain periods of sensitivity to special environmental stimuli that affect growth (Edwards, 2006). The long-term athlete development (LTAD) model suggests that children and adolescents during the sensitive period are more sensitive to training-induced adaptation (Balyi and Hamilton, 2004). Studies showed the first sensitive period for developing linear, lateral, and multi-directional speed (i.e., COD) occurs between the ages of 6 and 8 for girls and between 7 and 9 for boys (Balyi and Hamilton, 2004; Balyi and Way, 2005; Rumpf et al., 2012). If children fail to develop these skills during this period, young players might lose the opportunity to reach their full potential (Balyi and Hamilton, 2004). On the contrary, research shows that most fitness parameters are trainable throughout childhood and should not be restricted to sensitive period at different stages of development (Ford et al., 2011; Rumpf et al., 2012). For instance, Rumpf et al. (2012) suggest that prepubertal children (male: 7–12 years; female: 7–11 years) gain more speed performance improvements from high levels of neural activation training (e.g., sprint training). Also, children at this stage possess better neuroplasticity, and the development of sport-specific movements including COD performance is necessary at an early childhood (Lloyd et al., 2013). Lloyd et al. (2013) suggested that both children and adolescent athletes should receive training program including fundamental movement skills (FMS), COD and reactive agility training (RAT), but the percentage of time should be allocated according to different stages (e.g., prepubertal stage: 60% FMS training, 25% COD training and 15% RAT; circumpubertal stage: 30% FMS training, 40% COD training and 30% RAT). These may support the concept of a sensitive period in which sprint and COD training should be trained throughout childhood, but different training adaptations predominate differently. In addition, most studies also show that 6–12 years old is the golden period of motor skill learning and motor development, which is often referred to as the sensitive or critical period of motor skill learning and motor development (Hirtz and Starosta, 2002; Watanabe et al., 2007; Ireland et al., 2019; Solum et al., 2020). For example, one study showed that musicians who started training early showed better performance on tasks (keystroke accuracy and response synchronization), which could be due to the fact that children’s neurodevelopment is not fully mature and the brain has greater plasticity during sensitive period of motor skill learning and motor development (Knudsen, 2004; Watanabe et al., 2007). It was considered that brain plasticity was likely highest in childhood and gradually decreased thereafter (Johansson, 2004). The potential reasons could be that in early childhood increased myelination in motor pathways leads to reduced reaction time and increased motor speed, which is associated with improved motor skills (Penhune, 2011). In addition, children develop stronger synapses between nerve cells as well as getting rid of those that are less adaptive (Knudsen, 2004). Overall, during the sensitive period of motor skill learning and motor development, the nervous system is particularly sensitive to related stimuli and is more prone to change when stimulated (Penhune, 2011). The brain develops new neural circuits during sensitive periods, laying the groundwork for later learning. Therefore, 7–8 years old seems to be a sensitive period for children’s motor development and motor learning, and targeted training should be able to better promote the improvement of fitness.

At present, some of the research focused on determining which training interventions can maximize speed and COD performance in young tennis players. These interventions are mainly NMT (Salonikidis and Zafeiridis, 2008; Barber-Westin et al., 2010, 2015; Fernandez-Fernandez et al., 2016, 2018). NMT is a complementary training program that includes both general (e.g., fundamental movements) and specific (e.g., exercises targeted to motor control) strength and conditioning activities, such as resistance, dynamic stability, core focused strength, plyometric and agility, that are designed to enhance health and skill-related components of physical fitness (Myer et al., 2011). It is a critical intervention for adolescents with high plasticity, starting in mid-childhood (approximately 7–10 years of age) and continuing throughout adolescence (Myer et al., 2011). For example, Fernandez-Fernandez et al. (2016) performed neuromuscular training (e.g., COD drills training, chasing training and ladder drills training) for 5 weeks before regular tennis training and observed a significant improvement in tennis players’ (12.9 ± 0.4 years) speed (5–20 m sprint) and COD (5-0-5 agility test) performance. In addition, Salonikidis and Zafeiridis (Salonikidis and Zafeiridis, 2008) compared the effects of plyometric training (PT), tennis-specific training, and combined training (PT combined tennis-specific training) on tennis players (21.1 ± 1.3 years), and observed that combined training (e.g., sprint training, repetition COD training) combined the advantages of both regimens and significantly improved speed (4 and 12 m sprint) performance compared to PT and tennis-specific training alone. Similarly, Fernandez-Fernandez et al. (2016) observed that adding PT (e.g., jump training and multidirectional movements) to regular tennis training resulted in a significant improvement in 10-m sprint time and COD (5-0-5 agility test) performance compared to tennis training alone (12–13 years). Generally, most studies have shown that NMT training (e.g., sprint training, multidirectional movements) combined with tennis specific training can significantly improve sprint and COD performance in 12–21 years old tennis players. However, to our knowledge, no studies have examined whether a combination of reglue tennis training and NMT for tennis beginners aged 7–8 has a similar effect.

Therefore, the purpose of this study was to compare the effects of an 8-week NMT combined with a regular tennis training program on the sprint and COD ability of tennis beginners aged 7–8 to a control group taking only regular tennis training. It was hypothesized that NMT combined with regular tennis training could produce greater improvements in sprint and COD ability among tennis beginners 7–8 years old when compared with regular tennis training only.

## Materials and Methods

### Study Design

This study was designed to compare the effects on tennis beginners of 8 weeks of NMT combined with regular tennis training for sprint and COD ability. Sprint and COD ability tests were carried out before (pre-test) and after training (post-test), including: (a) 30 m sprint test, (b) 3 × 10 shuttle run test, (c) 5–10–5 test, and (d) spider agility test.

### Participants

Thirty-two participants were randomized into a training group (TG: eight females and eight males) and a control group (CG: eight females and eight males; see Table 1). The inclusion criteria were as follows: (1) no previous participation in tennis lessons, NMT and (2) no physical or mental defects or congenital diseases. The TG underwent a training program that incorporated tennis lessons, NMT between 15:30 and 17:00 (at a tennis court) on Mondays and Tuesdays for eight continuous weeks. The CG underwent only tennis-specific training from 15:30 to 17:00 on Wednesdays and Thursdays for eight continuous weeks. All participants completed two familiarization sessions before baseline measurements were taken (i.e., pre-test). All participants signed informed consent forms with the help of parents/teachers and were informed that they could withdraw from the experiment at any time. The study was approved by the Human Research Ethics Committee of Shanghai University of Sports.

**Table 1:** Demographic characteristics of the participants (Mean ± SD).

	TG (*n* = 16)		CG (*n* = 16)		Females (*n* = 8)	Males (*n* = 8)	Females (*n* = 8)	Males (*n* = 8)
Age (years)	7.4 ± 0.5	7.2 ± 0.4	7.2 ± 0.5	7.2 ± 0.4
Height (m)	1.19 ± 0.48	1.19 ± 0.57	1.19 ± 0.51	1.21 ± 0.44
Body mass (kg)	21.0 ± 2.71	21.3 ± 2.91	21.5 ± 2.76	22.3 ± 2.63

TG, training group; CG, control group.

### Measurements

#### Speed Tests

##### 30 m Sprint Test

The 30 m sprint test was used to assess straight line acceleration ability. After a warm-up, participants were instructed to sprint as fast as possible from a standing start just behind the Smartspeed laser timing gate system (Fusion Sports, Inc., Milton, Australia). Time was automatically recorded by the timing gate system. The participants each performed two trials with 2 min of rest in between, and each participant’s best performance was used for subsequent statistical analysis.

##### 3 × 10 m Shuttle Test

Each participant sprinted from the start line, completely crossed the finish line (10 m away from the start line), then turned 180° to sprint back to the start line, followed by another immediate sprint back to the finish line. Total time was automatically recorded using the Smartspeed laser timing gate system. Two trials with 2 min of rest in between were conducted, and each participant’s shorter times were recorded for subsequent analysis.

#### COD Tests

##### 5–10–5 Test

Three marker barrels (A, B, and C) were placed five yards apart on a track and field court. The participants started at the first marker barrel (A), and upon command, sprinted left and touched the second marker barrel (B); the participant then turned 180° and sprinted to touch the far-right marker barrel (C), followed by another sprint back to the first marker barrel (A; Rossi et al., 2017). Time was automatically recorded using the Smartspeed laser timing gate system. The participants performed two trials separated by 3 min of rest. The faster trials of each participant were used for subsequent data analysis.

##### Spider Agility Test

Five tennis balls were placed on the sidelines and along the service line. Participants started from the middle of the baseline (M), ran to pick up a ball, and ran back to leave the ball at M. Each ball was retrieved in a clockwise manner. The time when the last ball was placed at M was recorded in seconds to two decimal places with a Tianfu stopwatch timer (Fuhai Chemical Glass Instrument Co., Ltd., Shenzhen, China; Kramer et al., 2016, 2017). The participants each performed two trials separated by a 5 min rest. The shorter times for each participant were used for subsequent data analysis.

### Training Program

The TG program included training in sprints, coordination, and COD in addition to the tennis lessons (see Table 2). Considering the young age of the participants and the complexity of the training contents, the strength and conditioning coach organized one meeting every 2 weeks to introduce the methods of training progression such as increasing the difficulty of the movements, changing the movements from single direction to multi-direction, and increasing the training load every 2 weeks. Proper techniques were demonstrated by the strength and conditioning coaches and maintained *via* verbal cues. The extra training for the TG was conducted as a 15–20 min component of the twice-weekly 90-min tennis classes. Each member of the TG strictly followed the training program and completed all sessions.

**Table 2:** Eight-week neuromuscular training program.

Week	Exercise activities	Sets (*n*)	Reps (*n*)	Rests (sets)	Exercises (*n*)
1–2	5 m sprint	1	2	1 min	2
	2 in forward	1	4	1 min	2
	2 in lateral	1	4	1 min	2
	Frontal 2 in 2 out	1	4	1 min	2
	Later 2 in 2 out	1	4	1 min	2
	Sprint back and forth	1	2	1 min	2
	Left sideline shuttle run	1	2	1 min	2
	Right sideline shuttle run	1	2	1 min	2
	Hexagon jump(40 cm)	4	2	1 min	2
3–4	10 m sprint	1	2	1 min	2
	1 lateral (back and forth)	1	4	1 min	2
	Lateral lckey shuffle	1	4	1 min	2
	Side step	1	4	1 min	2
	Crossover step	1	4	1 min	2
	Sprint off left sideline in forward	1	2	1 min	2
	Sprint off right sideline in forward	1	2	1 min	2
	Hexagon jump (40 cm)	4	2	1 min	2
5–6	20 m sprint	1	2	1 min	2
	Frontal 2 in 2 out forward	1	4	1 min	2
	Frontal 2 in 2 out backward	1	4	1 min	2
	Frontal 1 in 1 out forward	1	4	1 min	2
	Frontal 1 in 1 out backward	1	4	1 min	2
	Throwing and catching balls over the net	1	4	1 min	2
	Four-corners run	1	2	1 min	2
	Hexagon jump (50 cm)	4	2	1 min	2
7–8	20 m sprint	1	2	1 min	2
	Rope ladder drills	1	4	1 min	2
	Throwing and catching balls over the net	1	4	1 min	2
	Four-corners run	1	2	1 min	2
	Hexagon jump (60 cm)	4	2	1 min	2

CG only attended 90-min tennis instruction (e.g., small steps in place with a racket, combination of small step and split step, swing practice in multi-directions, etc.) twice a week for eight continuous weeks.

### Statistical Analyses

Descriptive statistics (mean ± SD) for the different variables were conducted. The Kolmogorov–Smirnov test and the Levene test for equality of variances were used to determine normal distribution. Two-way repeated measures analysis of variance (ANOVA) were used for data analysis [2 time-points (pre-test vs. post-test) *two group (TG vs. CG)]. Partial eta squared (η^2^) was used as the effect size (ES) estimation for the time by group interaction effect with its strength being interpreted as the following: small < 0.06; moderate < 0.14; large ≥ 0.14 (Cohen, 1988). The level of significance was set at *p* < 0.05. The sphericity of the data was validated by Mauchly’s W statistic. If this test were violated, the Greenhouse–Geisser correction would have been used for adjustment.

## Results

**Figure 1:**
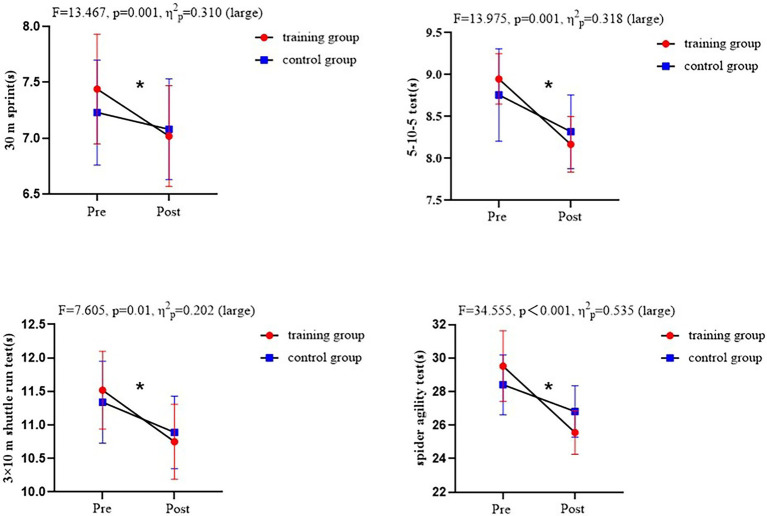
Comparison of training group and control group pre-and post-intervention. *Significant difference from pre-test within the group.

No significant differences were observed between the TG and CG in any of the variables analyzed pre-intervention. Significant differences in the interaction between time and group were found in the following: 30 m sprint test (*F* = 13.467, 95% CI = 7.163–7.506, *p* = 0.001, *η*^2^*_p_* = 0.310); 5–10–5 test (*F* = 13.975, 95% CI = 8.696–9.017, *p* = 0.001, *η*^2^*_p_* = 0.318); 3 × 10 m shuttle run test (*F* = 7.605, 95% CI = 11.213–11.642, *p* = 0.01, *η*^2^*_p_* = 0.202); and spider agility test (*F* = 34.555, 95% CI = 28.258–29.670, *p* < 0.001, *η*^2^*_p_* = 0.535; see Figure 1).

## Discussion

The current study aimed to investigate the effects of an 8-week NMT combined with regular tennis training for beginning tennis players aged 7–8. To our knowledge, this is the first study conducted in 7–8 years old tennis beginners about NMT combined with regular tennis training. Significant improvements were observed in speed (30 m sprint test and 3 × 10 m test) and COD ability (5–10–5 test and spider agility test) for the TG, demonstrating the importance of NMT combined with regular tennis to speed and COD ability in tennis beginners during the first sensitive period. The results suggest that NMT combined with regular tennis training appears to be an effective method of improving these abilities in this particular age group.

The speed (30 m sprint test and 3 × 10 m test) and COD (5–10–5 test and spider agility test) performances improved for the TG as a result of the 8-week training program intervention when compared to the CG. These differences may be related to the lack of sufficient training stimulation to increase neural drive in CG. Due to the size of the court and the specific technique movements only in regular tennis training, children are rarely exposed to sprint distances over 10 m and COD training. Therefore, NMT combined with regular tennis training may provide an additional advantage when compared to regular tennis training. In addition, the improvement of various neuromuscular adaptations in children may be the important factor for the enhancement of sprint and COD performance after NMT combined with regular tennis training. Preadolescence (male: 7–12 years; female: 7–11 years) is considered to be a period of rapid development (Borms, 1986) and high plasticity of children’s neuromuscular system (Ford et al., 2011). Studies have shown that children (7–8 years old) benefit most from training related to neural adaptation (Fernandez-Fernandez et al., 2016). The structure or function of the brain is sensitive to specific external stimuli and more likely to establish nervous motor circuits (Thomas and Johnson, 2008). NMT is a vital intervention program for children with high plasticity of cognitive and motor capabilities (Kraemer et al., 1989). The NMT program in this study mainly includes sprinting (e.g., 5–20 m sprint), coordination training (e.g., ladder drills), and multidirectional movements (e.g., four-corner run). It can effectively improve children’s neural adaptation, such as an increase in motor unit activation and synchronization, as well as an increase in motor unit recruitment and excitation frequency (Baechle and Earle, 1994).

The 30 m sprint and 3 × 10 m shuttle sprint performances improved for the TG as a result of the 8-week training program intervention when compared to the CG. Van Praagh (1998) suggested training methods that aim to improve coordination, movement efficacy, or velocity should be used to optimize the effectiveness of speed training before puberty. Speed is reflected in linear sprints. Speed performance may be highly influenced by technique (Faigenbaum et al., 2007), balance (Chaouachi et al., 2014), anthropometrics (height, body mass, etc.; Meyers et al., 2017), motor performance, and coordination (Behringer et al., 2011). An increase in coordination and a decrease in sprint time may have been important factors for improved speed in the current study. The TG NMT program incorporated sprint training and intermuscular coordination training, such as 5–20 m sprint and some ladder drills training. The evidence suggests that the most efficient training method for improving sprint performance is sprint training (Padrón-Cabo et al., 2020). Sprint-based activities, such as sprinting 5–20 m at maximum speed, involved heavy use of stretch-shortening-cycle movements, and thus may increase rate of force development, impulse production and stiffness of muscles which are associated with optimal sprint performance (Haff and Triplett, 2006). Sprinting is divided into three phases: initial acceleration, transition, and maximum velocity phases (von Lieres Und Wilkau et al., 2020). Sprint training may increase the velocity of the acceleration phase, resulting in significant decreases in ground contact time, which may allow children to achieve increases in stride rate and thus improve sprint performance (Kotzamanidis, 2006). One study indicated that a 10-week sprint training program could improve 20 m sprint performance for 11-year-old children (Kotzamanidis, 2006). A study by Venturelli et al. (2008) also reported improvements in 20 m sprint performances for male soccer players following 12 weeks of sprint training. In addition, coordination training (e.g., ladder drills training, hexagon jump) also seems to be a suitable method for improving speed performance (Venturelli et al., 2008; Padrón-Cabo et al., 2020), possibly providing a greater neural stimulus that results in better intramuscular and intermuscular coordination, and thus improvement in speed performance.

The 5–10–5 and spider agility tests also showed improved COD performance for the TG over 8 weeks compared to the CG. Our training program incorporated coordination exercises (e.g., ladder drills and hexagon jump) and multi-directional training (e.g., four-corner run) to improve intermuscular coordination, as well as acceleration and deceleration ability. Coordination training may improve intermuscular coordination and provide better synchronization of body segments to effect an improvement in COD ability. Multi-directional training may improve the speed and force of muscle contraction (e.g., eccentric strength) and enables the players to switch between deceleration and acceleration motions. This is critical for COD performance and another factor that affects COD ability (Balyi and Way, 2005; Brughelli et al., 2008; Barber-Westin et al., 2015). Meanwhile, the improvement of linear sprint speed may also contribute to the improvement of children’s COD performance. Improving the sprint speed in short distance can benefit the initial and exit velocity in COD (Vanrenterghem et al., 2012). However, the contribution of sprint speed to COD performance may vary with the magnitude and amount of angle changed during a number of the direction changes (Young et al., 2002). Pettersen and Mathisen (2012) reported that the COD performance of 11–12 years old boys were significantly improved after 6 weeks of short distance sprint training, and also found that there was a significant transfer effect between the performance of straight-line sprint and COD performance (*r* = 0.68–0.75).

The significant improvement of sprint and COD ability may help children improve motor competence and physical fitness. On the one hand, the improved sprint and COD ability can help children further enhance their performance in tennis competitions. It also can help children achieve faster directional transitions, reach a point on the court faster and/or answer to a tennis ball faster. On the other hand, it provides a better basis for children to use targeted strength and conditioning training program to improve their physical fitness in tennis. Based on the current findings, improved speed and COD performance may help athletes achieve better long-term development and provide more lasting benefits, such as improved physical fitness, tennis skills and competitive performance. However, to know whether there are long term benefits of participating in such a training program was beyond the scope of current research, and future research should be conducted to investigate this issue. It would be useful if the same group of children were followed up some time after the intervention to check whether the positive changes reported here were maintained.

## Conclusion and Limitations of the Study

### Conclusion

NMT combined with regular tennis training program could produce greater improvement in sprint and COD ability among sensitive period childhood tennis beginners when compared to regular tennis training intervention only. In view of the current results, when tennis coaches design training programs for children during the first sensitive period, it is recommended that specific tennis NMT be added to the program to improve speed and COD ability.

### Limitations of the Study

A limitation in the current study that should be addressed is that it did not explore the underlying mechanisms of improved speed and COD, such as neuromuscular and lower limb force changes, after the training program. Therefore, further study is needed to examine the effects of neuromuscular adaptation and lower limb force following NMT combined with regular tennis training program.

### Practical Applications

Researchers have found similar benefits of NMT combined with tennis training on sprint and COD performance in other age groups of 12–13 and 21 years old. However, to our knowledge, this is the first study conducted in 7–8 years old tennis beginners about NMT combined with regular tennis training. Overall, our results demonstrate the potential value of incorporating an injuries-free, time-saving, inexpensive and developmentally appropriate NMT program into regular tennis training for children aged 7–8 years. Meanwhile, the results extend previous research work advocating the use of NMT program in children (e.g., 8–12 years), confirming that participation in the NMT program combined with regular tennis training was an effective approach to better improve sprint and COD performance of children aged 7–8 years. The performance improvements shown in current study are of great interest to tennis coaches and are directly applicable to childhood tennis beginners aged 7–8 years. It is recommended that tennis coaches implement NMT to improve players’ performance. The results of the study could help coaches and sports scientists develop better guidelines and recommendations for childhood tennis beginners’ training prescription and competition preparation.

## Data Availability Statement

The original contributions presented in the study are included in the article/supplementary material, further inquiries can be directed to the corresponding author.

## Ethics Statement

The studies involving human participants were reviewed and approved by the Human Research Ethics Committee of Shanghai University of Sports. Written informed consent to participate in this study was provided by the participants’ legal guardian/next of kin.

## Author Contributions

Z-HW, R-CP, and M-RH performed all the experiments. DW designed the experiments. Z-HW and DW analysed the experimental results. Z-HW wrote the manuscript. All authors reviewed and approved the submission of the manuscript.

## Funding

This research was funded by a grant from Program for Overseas High-level Talents at Shanghai Institutions of Higher Learning under Grant No. TP2019072, the Natural Science Foundation of the Higher Education Institutions of Jiangsu Province under Grant No. 17KJB320008, and Shanghai Key Lab of Human Performance (Shanghai University of Sport) under Grant No. 11DZ2261100.

## Conflict of Interest

The authors declare that the research was conducted in the absence of any commercial or financial relationships that could be construed as a potential conflict of interest.

## Publisher’s Note

All claims expressed in this article are solely those of the authors and do not necessarily represent those of their affiliated organizations, or those of the publisher, the editors and the reviewers. Any product that may be evaluated in this article, or claim that may be made by its manufacturer, is not guaranteed or endorsed by the publisher.
